# Non-Compliance with Growth Hormone Treatment in Children Is Common and Impairs Linear Growth

**DOI:** 10.1371/journal.pone.0016223

**Published:** 2011-01-31

**Authors:** Wayne S. Cutfield, José G. B. Derraik, Alistair J. Gunn, Kyle Reid, Theresa Delany, Elizabeth Robinson, Paul L. Hofman

**Affiliations:** 1 Liggins Institute, University of Auckland, Auckland, New Zealand; 2 National Research Centre for Growth and Development, University of Auckland, Auckland, New Zealand; 3 Departments of Physiology and Paediatrics, University of Auckland, Auckland, New Zealand; 4 Pharmaceutical Management Agency of New Zealand (PHARMAC), Wellington, New Zealand; 5 Department of Epidemiology and Biostatistics, University of Auckland, Auckland, New Zealand; University of California, Los Angeles, and Cedars-Sinai Medical Center, United States of America

## Abstract

**Background:**

GH therapy requires daily injections over many years and compliance can be difficult to sustain. As growth hormone (GH) is expensive, non-compliance is likely to lead to suboptimal growth, at considerable cost. Thus, we aimed to assess the compliance rate of children and adolescents with GH treatment in New Zealand.

**Methods:**

This was a national survey of GH compliance, in which all children receiving government-funded GH for a four-month interval were included. Compliance was defined as ≥85% adherence (no more than one missed dose a week on average) to prescribed treatment. Compliance was determined based on two parameters: either the number of GH vials requested (GHreq) by the family or the number of empty GH vials returned (GHret). Data are presented as mean ± SEM.

**Findings:**

177 patients were receiving GH in the study period, aged 12.1±0.6 years. The rate of returned vials, but not number of vials requested, was positively associated with HVSDS (p<0.05), such that patients with good compliance had significantly greater linear growth over the study period (p<0.05). GHret was therefore used for subsequent analyses. 66% of patients were non-compliant, and this outcome was not affected by sex, age or clinical diagnosis. However, Maori ethnicity was associated with a lower rate of compliance.

**Interpretation:**

An objective assessment of compliance such as returned vials is much more reliable than compliance based on parental or patient based information. Non-compliance with GH treatment is common, and associated with reduced linear growth. Non-compliance should be considered in all patients with apparently suboptimal response to GH treatment.

## Introduction

Non-compliance with prescribed drug treatments is widespread [1]. Key factors include discomfort (e.g. associated with daily injections) [2], long-term treatment [3], complexity of treatment regimens, age, individual and family dynamics, as well as patient or family's understanding of treatment benefits and consequences of non-compliance [3], [4]. These principles suggest that children administered growth hormone (GH) as a daily subcutaneous injection for many years are at risk of treatment non-compliance. GH treatment is very expensive; the annual cost for a 30 kg child has been estimated as US$ 15,000 to US$ 20,000 per annum, while the annual costs of treating adolescents to maximize adult height can reach US$ 50,000 [5]. Thus, non-compliance with treatment must lead to appreciable waste of funding; it is presumed that it would also impair long-term linear growth [6], [7], but there are few objective data.

In this study we examined compliance with GH treatment and its effect on linear growth over a single four-month period in a complete national cohort of New Zealand children and adolescents. In view of the reliance of previous studies on self-report or prescription data [6]–[11], we contrasted a caregiver-reported measure of GH utilisation with an objective measure (number of GH vials returned during the interval).

## Materials and Methods

Review with the Multiregion Ethics Committee (Wellington, New Zealand) determined that ethics approval of this comprehensive audit was not required.

This study was an unbiased, anonymized national survey of GH compliance in all New Zealand children and adolescents receiving publically-funded GH in a single four-month interval in 2007. All patients receive monthly allocations of GH based upon surface area dosing. Each month, the caregivers of the patients are contacted by phone to determine how many residual vials of GH the family have, and thus how many vials are required for the coming month (GHreq). For the duration of this review, caregivers were also asked to return all empty vials of GH to the growth hormone administrator each month, using a prepaid courier bag sent directly to the family (GHret). Parents were actively encouraged, but could not be mandated to return all empty vials. To ensure that no empty GH vials were left over from periods preceding the study, empty vials were requested starting the month before the study.

Compliance was evaluated in two ways: number of GH vials required each month (GHreq; based on verbal self-report by the patient's caregivers) and number of used GH vials returned (GHret), with both expressed as a percentage of the number of vials prescribed for each patient. Based on the known doses of GH prescribed, no patients should have used less than one vial of GH over the 4-month duration of this investigation, and therefore patients who returned no vials over the study period were excluded from analysis.

Growth data are routinely obtained every 6 months for all patients. Growth rates were calculated for the 6–8 month-period that encompassed the interval of this audit. Changes in height were recorded and converted to height velocity standard deviation scores (HVSDS) to control for sex and age differences. For the purposes of analysis, compliance was defined as 85% or better adherence to the prescribed treatment, equivalent to an average of one missed dose a week. Patients were divided into three groups according to their rate of compliance: High compliance (missed ≤1 dose a week on average), Medium (missed >1 and <3 doses/week), and Low (missed ≥3 doses/week).

The association between GHret and GHreq to HVSDS was assessed using non-parametric Spearman's rank correlations. HVSDS was compared across compliance groups using a general linear regression model (GLM), which also included as factors diagnosis (i.e. KIGS code 1, 2 or 3) and an interaction between diagnosis and compliance group, in order to account for differential growth responses to GH treatment. Subsequently, the effects of age, sex, ethnicity, area of residence (cities (>100,000 inhabitants) vs. towns and rural areas) and diagnosis on GH compliance for each subject were examined. A GLM was used with compliance rate as the response, and sex, ethnicity, diagnosis and area of residence as independent variables, and age included as covariate. A binary logistic linear regression was then adopted to determine if any of the above factors had an effect on whether a patient was compliant or not. Posthoc pairwise comparisons were carried out using Tukey's simultaneous tests. All analyses were carried out in Minitab (Minitab v.15, Pennsylvania State University, USA), with Johnson transformation as required to stabilize the variance. Data are presented as mean ± SEM.

## Results

175 patients received GH in the 4 month interval of the analysis and were 12.1±0.6 years old with 48% males. Diagnoses included GH deficiency (57%), Turner Syndrome (27%), idiopathic short stature (7%), small for gestational age (6%), Prader Willi Syndrome (3%) and other (6%). The caregivers identified the children as European & other (75%), Maori (15%), Asian (7%), and Pacific Islander (4%). 35% of patients resided in Auckland, 34% elsewhere in the North Island, and 22% in the South Island.

**Figure 1:**
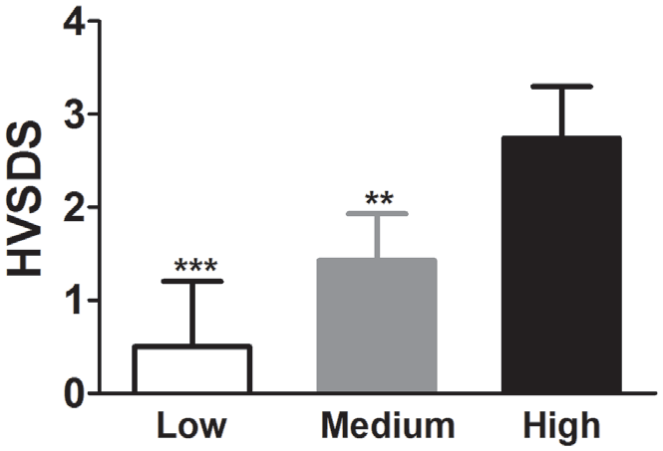
Height velocity standard deviation scores (HVSDS) over 6–8 months according to the level of compliance with GH treatment: High (*n* = 30) missed ≤1 dose/week, Medium (*n* = 51) missed >1 and <3 doses/week, and Low (*n* = 29) missed ≥3 doses/week. Data are mean ± SEM. **p<0.01, ***p<0.001 vs High.

A total of 25 patients failed to return a single vial during the study period, and were excluded from the GHret analysis. The overall estimated rate of non-compliance (i.e. patients missing more than one injection per week) was 66% (73/110) according to GHret and 34% (59/172) according to GHreq. HVSDS was positively correlated with the rate of returned vials (ρ = 0.20; p<0.05) but not with GHreq. Further, average linear growth of patients increased as GHret compliance levels increased, and missing more than one dose a week significantly reduced linear growth (p<0.01) (Fig. 1), whereas GHreq showed no association with growth (p = 0.83). GHret was therefore used for subsequent analyses. Finally, patients with organic GH-deficiency tended to grow faster than subjects with other causes of short stature (HVSDS 2.32±0.60 vs 0.93±0.38; p<0.06).

Compliance rates were not associated with age, gender, diagnosis or area of residence. However, Maori patients missed nearly one extra GH dose a week on average in comparison to other ethnic groups (p<0.05).

## Discussion

This national survey indicates that non-compliance with GH treatment is very common in New Zealand, based on objective data, in a setting where there is regular, direct contact with caregivers and no personal financial constraints on GH uptake. Further, the two thirds of patients who on average missed one or more doses of GH each week showed significantly reduced linear growth compared with compliant patients. To our knowledge our study is the first to provide robust evidence that non-compliance is a common cause of suboptimal response to GH treatment in children and adolescents.

The use of returned GH vials in this study provided an objective measure of compliance in a complete population of children and adolescents receiving GH. A higher GHret should reflect the number of vials used in the previous month and thus denote greater compliance. A potential limitation is that return of used GH vials was not a mandatory pre-condition to receive new vials. Therefore, although GHret was an objective measure in contrast with caregiver-reported GHreq, vials might have been broken, lost, or the empty vials simply not sent back to the programme, so that GHret could have underestimated compliance. Nonetheless, the strong correlation with patient growth suggests that this approach still provided a reasonably valid measure of compliance. An alternative approach would be the use of electronic injection devices with a dosage counter to accurately assess patient compliance. Such devices are available but are limited by cost, complexity and robustness. This may become practical for individual care as technological advances lead to smaller, robust and cheaper electronic injection devices.

In contrast, in the present study compliance estimated by caregiver-report showed no correlation with growth response. This method is broadly similar to retrospective patient questionnaires or GH prescription data used in previous studies [6]–[11]. Although a higher GHreq should also indicate better compliance, caregivers may not admit to having failed to use the appropriate amount of GH, and therefore GHreq is likely to overestimate compliance. In other conditions, consistent with our findings, such studies are reported to overestimate adherence to drug treatment, whether due to forgetfulness, embarrassment or fear of recrimination [12]. Our study indicates that patient and/or parent reported compliance (GHreq) is an unreliable measure of GH treatment compliance. This is consistent with the finding that in children requiring antibiotic prophylaxis after urinary tract infection, 97% of parents reported giving antibiotics every day, but only 69% of urine samples were positive [13]. Understanding compliance is important as clinicians are generally unable to accurately predict treatment adherence [14], and non-compliance can mislead medical practitioners regarding the efficacy of treatment and lead to invasive testing for other causes of poor growth.

Further, compliance is not an end in itself. Only one previous study (reported solely in abstract form) has examined the quantitative relationship between compliance and linear growth [7]. The present study demonstrates for the first time that missing more than one GH dose a week can compromise longitudinal growth. Importantly, we showed a continuous relationship between missed doses and growth. In other words there was no apparent threshold effect, and missing more than one GH dose each week would likely affect growth.

There are few validated strategies to improve adherence to GH treatment. Reduction of injection frequency with depot GH would likely improve compliance, as seen with other depot injectable therapies such as GnRH agonists [15], but is not possible at present. Since the pain of injections has been reported to be a concern [2], strategies to reduce pain such as automatic pens or use of finer injection needles may be beneficial. Oyarzabal *et al* showed in a multicentre survey that users of automatic pens were significantly more compliant with GH treatment than those using conventional syringes [8]. Although there are no data on GH, in other settings daily drug reminder charts [16], financial incentives [17], [18], provision of enhanced support and education for patients and their families on the benefits of therapy and consequences of non-compliance may improve adherence rates [19]. However, parents receive relatively consistent support and education in New Zealand, both centrally, at the time of induction and around GH deliveries, and from paediatricians and clinic nurses, and thus improved education is unlikely to suffice.

The finding of a higher incidence of non-compliance among the indigenous Maori community is of concern. Maori children have a higher burden of many diseases as well as higher rates of complications during treatment of other chronic illnesses such as diabetes [20], [21]. Previous authors have acknowledged that Maori also display poorer compliance with medical treatments [21], so that developing culturally safe ways to improve adherence to long-term treatment regimens will be critical to improving outcomes in vulnerable groups [20].

In conclusion, the present study strongly suggests that poor-compliance is one of the most common factors underlying suboptimal growth during GH therapy, and should be actively evaluated and managed before considering other illnesses that can affect growth or partial GH resistance. It is critical to find ways to improve families' compliance with GH treatment.
